# Royal Orthopædic Hospital

**Published:** 1902-10-04

**Authors:** Richard B. Martin, H. H. Marks, Ernest Flower

**Affiliations:** President; Treasurer; Chairman of Committee; Hon. Secretary


### Editor's Letter-Box.

[Our Correspondents are reminded that prolixity is a great bar to publication, and that brevity of style and conciseness of statement greatly facilitate early insertion.]

### ROYAL ORTHOPÆDIC HOSPITAL.

To the Editor of *The Hospital.*

As considerable misapprehension appears to exist in some quarters regarding the scheme for rebuilding this hospital upon a new site, and the matter has lately been much discussed in the medical press, we feel that, in the interest of all concerned in the welfare of the Charity, some official statement should be made in order that the main points which have influenced the authorities may be fully understood.

Critics of the policy adopted by the hospital authorities contend—(1) that there is no pressing need to rebuild; (2) that when we rebuild we should do so on the present freehold site, which is too valuable an asset to be parted with at price agreed upon; and (3) that the proposed sale and rebuilding elsewhere will not benefit the hospital financially.

With regard to (1) the position of the authorities is as follows:—The present buildings were never intended for a hospital, and are therefore most inconvenient. Amongst many other wants there is no proper accommodation for the staff; there are no ward bath-rooms and no proper antiseptic operating theatre with the various appliances and conveniences required in modern hospitals. These and other matters have provoked the criticism of experts, including the Council and Visitors of the King Edward's Hospital Fund. In fact, affairs were so bad that in 1899 nearly £2,000 had to be spent in improving the sanitary arrangements and providing a new drainage system. This outlay was absolutely necessary at the time, unless the hospital had closed its doors; but no amount of expenditure could make the buildings fit for the purposes of the Charity.

Much time and careful consideration have been given to the point (2). The question of rebuilding was first considered in 1897, when the committee, not wishing to rebuild elsewhere unless compelled to by want of space, had sketch plans prepared to see what accommodation could be provided upon the existing site; and it was found that it was impossible to erect a hospital of even the present number of beds (50) with all the modern requirements and in accordance with the highest standard of surgical science on our site of less than 7,000 super. feet, with frontages of 37 feet only, and an open space in the centre which could not be built over.

In 1898 the Prince of Wales' (now King Edward's) Hospital Fund did not make, as had been expected, an award to the institution, and it was subsequently ascertained that the Council did not approve of the character of the buildings in which the work of the Charity is conducted, and that, in their opinion, the hospital should be rebuilt without delay. In 1900 the visitors of the Fund made an inspection of the premises, and, as a result of their report, an award was made to be devoted towards rebuilding on a new site, the grant being conditional upon this being done and steps taken without delay.

At the end of last year the committee opened negotiations with a probable purchaser of the site, who has now offered to take it on a lease for 99 years, at £1,400 per annum, with the option to purchase within three years at £40,000, and favourable conditions for allowing time to acquire a new site, rebuild, &c., before relinquishing the present premises. This offer the trustees, the committee, and expert authorities consider favourable; and the Charity Commissioners after an exhaustive inquiry and a report by their own valuer have given it their approval.

The committee have secured the offer of a most suitable corner site from the Duke of Bedford, situate at the junction of Coram Street and Herbrand Street, near Russell Square, the chief frontage being in the thoroughfare which has a south aspect. The site, which is considerably larger and better shaped for building purposes than the existing one, has been offered on a 99 years' lease at a ground rent of £500 per annum, and in the opinion of the authorities and their professional advisers a good modern hospital can be erected in accordance with the requirements of present-day sanitary and surgical science.

Dealing with point (3) one newspaper has criticised the action of the authorities on the lines that the sale and changes of site will not result in any considerable financial advantage. We are fully alive to this. It should be clearly understood that the change is being made solely with the view of securing greater efficiency and more comfort for the inmates. The public is much more likely to support an institution which is efficient and up to date than one which is far from being so, and is therefore constantly open to severe criticism.

In conclusion we wish to say that we simply put this forward as a presentment of our case in consequence of the one-sided statements which have been appearing, and which might prejudice the hospital in the minds of those who have no knowledge of our reason for action. We have no intention of entering on any controversy, and are content with the knowledge that our policy has the full approval of the Charity Commissioners and an overwhelming number of the Governors of the hospital, who have expressed their entire approval of the scheme on three separate occasions.

We are, Sir, yours obediently,

Denbigh, President;
Richard B. Martin, Treasurer;
H. H. Marks, Chairman of Committee;
Ernest Flower, Hon. Secretary.

